# A Critical Assessment of the Quality and Readability of Information on Gastric Cancer Available on the Internet for Patients

**DOI:** 10.7759/cureus.71691

**Published:** 2024-10-17

**Authors:** Eoghan Burke, Patricia Harkins, Mayilone Arumugasamy

**Affiliations:** 1 Surgery, Royal College of Surgeons in Ireland (RCSI), Dublin, IRL; 2 Medicine, Royal College of Physicians of Ireland (RCPI), Dublin, IRL

**Keywords:** gastric cancer, health information, health literacy, health on the internet, human factors

## Abstract

Introduction

It is now commonplace for patients to consult the internet with health-related questions. Unfortunately, the quality of information provided to them online is highly variable. Ensuring that patients get high-quality, reliable information is essential for all pathologies. Gastric cancer (GC), with its often subtle early symptoms and signs, is one such pathology where early identification is crucial. Ensuring high-quality information availability online for GC is thus essential to increasing rates of early detection.

Aims

This study aimed to assess the quality and readability of information posted on websites related to GC.

Materials and methods

We applied the search term “gastric cancer” or “stomach cancer” to the top three search engines, namely Google, Yahoo, and Bing. Using predefined inclusion and exclusion criteria, we identified 20 unique websites posting information related to gastric cancer (GC). We then assessed the quality and readability of the information posted on these websites. We used recognized tools to complete these assessments, including the JAMA benchmark criteria, the DISCERN tool, the Flesch Reading Ease score (FRES), and the Flesch-Kincaid Grade Level (FKGL). We also developed and used a novel GC-specific content assessment tool. Furthermore, we assessed whether or not each website was awarded the Health on the Internet Seal of Approval.

Results

The average JAMA score was 1.55, with none of the twenty unique websites scoring the maximum 4 points. The average DISCERN score was 54.8 (68.5%), with no website achieving the maximum of 80. The HON seal was present in only six websites (30%). The average GCSCS score was 11, with only five websites achieving a maximum score of 13 (25%). The average FRES and FKGL were 52.7 and 9.7, respectively.

Conclusion

Our study underscores the critical need for more high-quality, reliable information about GC online. We also emphasize the importance of ensuring this information is comprehensible to most patients, as it directly impacts their health outcomes.

## Introduction

It is now commonplace for patients to turn to the Internet for health-related information. Indeed, approximately 7 million searches are performed daily on Google for health-related issues [1]. Furthermore, one study cited 72% of US adults researching health-related topics online in the previous 12-month period [2]. There are many benefits to accessing health-related information online, including ease and rapidity of access. 

However, it is widely reported that the quality of online health-related information is poor [3]. Interestingly, despite considerable concern about the quality of health-related information available online, Rainie et al. found that 52% of internet users who employed health-based websites felt that “almost all” or “most” of the information was credible [4]. This suggests an issue with the general public’s health literacy and ability to critically assess information on websites.

Ensuring patients access high-quality, reliable, and readable information is vitally important. It ensures that patients are knowledgeable about their condition and prompts them to seek medical attention when appropriate. False or misleading information may lead to delays in diagnosis and poorer health outcomes.

Ensuring a timely diagnosis of GC is vitally important. Early signs and symptoms of GC can be subtle. If not diagnosed early, the prognosis for GC is poor [5]. This again highlights the importance of having high-quality GC-related information online.

This study aims to critically assess the quality and readability of GC information posted online by evaluating the websites returned as top hits by the major search engines. Similar to other authors, we will determine the quality and readability of the information using predefined tools, but uniquely, we will employ the novel Gastric Cancer Specific Content Score. By conducting this research, we aim to increase both clinicians’ and the public’s awareness of the quality of GC-related information available online.

An earlier version of this study was presented in poster format at the 17th IFSES World Congress of Endoscopic Surgery on 24-27 November 2021 in Barcelona.

## Materials and methods

To identify websites to analyze, we searched the top 3 search engines (Google, Yahoo, and Bing) using the search terms “gastric cancer” and “stomach cancer." Google is widely reported to be the most commonly used search engine [6]. So, to reflect this, we analyzed the top 20 websites returned by Google and the top 10 returned by Yahoo and Bing, following a similar methodology employed previously by the authors [7]. 

Our searches were performed on the 20th of June 2024. We included websites that satisfied our inclusion criteria: all free, English-language websites that did not require a password or sign-up. To assess websites that are targeted at patients, we excluded any websites that were targeted at professionals. We then removed all duplicate websites to yield a list of unique websites, which would be analyzed further and in more detail. Two authors performed this task independently, and a third author resolved conflicts.

Each website was evaluated for quality using several recognized and validated tools and a new and novel gastric cancer-specific assessment tool. The tools used included the JAMA benchmark criteria, the DISCERN tool, the presence or absence of the Health On The Internet (HON) seal, and the GC Specific Content Score (GCSCS).

The two validated tools used to assess the quality of health-related information were the JAMA benchmark criteria and the DISCERN tool. The JAMA benchmark criteria were established in 1997 by William Silberg et al. [8]. They include four domains: authorship, attribution (clear referencing of material), disclosure (of all relevant conflicts of interest), and currency (dates the content was posted and updated). A score of 1 is assigned if the domain is addressed and 0 if not. Thus, the maximum score possible is 4, and the minimum score is 0. To demonstrate that information conveyed by a website is of reasonable quality, it should satisfy all domains of the JAMA criteria. 

To better assess the quality of the content of a website discussing health-related information, the DISCERN project, with the resultant DISCERN scoring tool and handbook, was developed in 1998. The tool aims to assess the reliability of the information and suitability of treatment choices offered by a medical website. There are 16 questions, scored on a 1-5 point scale, and a maximum score of 80 is available [9]. It is thus more rigorous than the JAMA tool in assessing the actual quality of information contained within a website, as distinct from the JAMA benchmark tool, which evaluates the integrity of the website's publication process.

A surrogate marker of the quality of information on a health-related website is whether or not the website was awarded the Health on The Internet Seal. This seal, provided by the Health On The Internet Foundation, a non-profit organization established in Switzerland in 1995, is a prestigious recognition of a website's commitment to providing accurate and reliable health information. Websites that agree to its principles and comply with its standards are awarded this seal, which strongly indicates the website's quality and reliability. Crucially, websites are not mandated by search engines to acquire this seal; instead, it is attained voluntarily. 

Acknowledging the specific drawbacks of each tool, the authors developed a novel tool explicitly targeted at the quality of health-related information about GC. The authors have published previously on a tool designed to assess the quality of information on oesophageal cancer [7]. The exact process was used to develop this gastric cancer-specific tool. The Gastric Cancer Specific Content Score (GCSCS) is a novel scoring tool developed by two authors interested in gastrointestinal surgery by referencing current peer-reviewed literature (Table 1). One point is allocated if the predefined concept is addressed, and zero points if it is not addressed. A maximum score of 13 points is attainable. 

**Table 1:** Gastric Cancer Specific Content Score (GCSCS). Thirteen questions patients commonly ask about GC. 1 point is awarded if the website mentions the topic, and 0 points are awarded if it is not.

Question Topic In Relation to Gastric Cancer	
Age Commonly Affected	
Sex Commonly Affected	
Pathophysiology	
Risk Factors	
Genetics	
H. Pylori	
Symptoms/Signs	
Diagnosis	
OGD Details	
Surgery	
Medical Management	
Endoscopic Treatment	
Prognosis	
Total Points available	13

Acknowledging the importance of health literacy in how patients interpret information on the website, each website was assessed for its readability using two validated tools. Namely, the Flesch Reading Ease Score (FRES) and the Flesch Kincaid Grade Level (FKGL) [10]. The FRES score assesses a passage's readability based on two key parameters: the number of words per sentence and the average number of syllables per word. The score runs from 0 to 100. It is widely accepted that from a health literacy standpoint, passages should score 70 or higher on the FRES [11]. 

The FKGL is a modification of the FRES that presents the readability of a passage at a grade level. Thus, it is designed to predict that the passage would be readable by a student of that education grade [12]. For health literacy, it is accepted that passages should be readable by an 8th-grade student [13].

## Results

**Figure 1:**
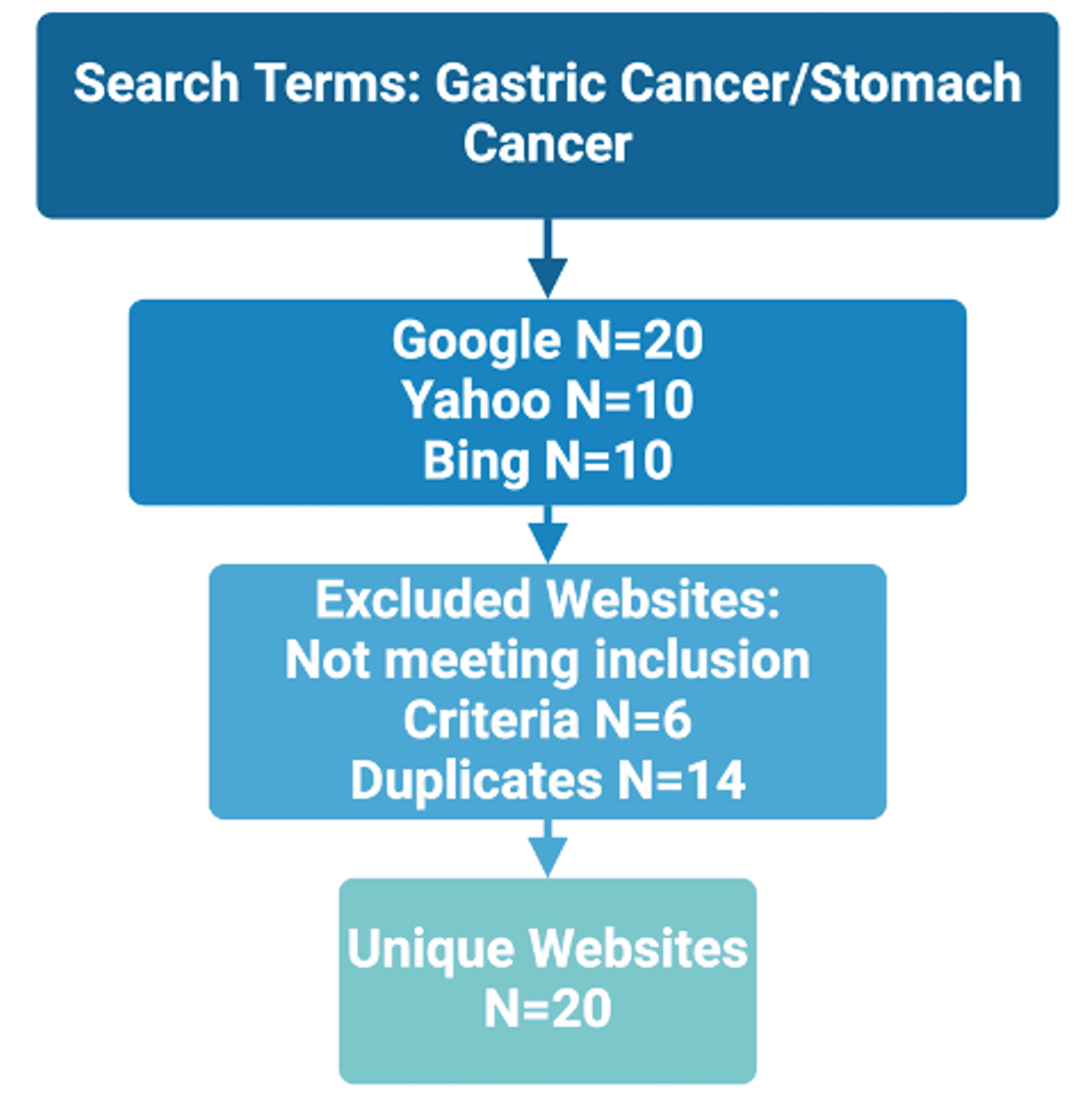
Internet search flow schematic. This schematic illustrates the process we followed to identify and select the websites for our study.

Our search strategy yielded twenty unique websites (Table 2). We excluded two academic papers, and four websites aimed at medical professionals and removed duplicate sites (n = 14), resulting in the final twenty unique websites (Figure 1). Google yielded 15 unique websites, with Yahoo and Bing yielding 3 and 2, respectively.

**Table 2:** Final list of unique websites.

Search Engine	Website Number	Website
Google n=15	1	https://www.cancer.gov/types/stomach/patient/stomach-treatment-pdq
	2	https://en.wikipedia.org/wiki/Stomach_cancer
	3	https://www.webmd.com/cancer/stomach-gastric-cancer#1
	4	https://www.cancer.org/cancer/stomach-cancer/detection-diagnosis-staging/signs-symptoms.html
	5	https://www.mayoclinic.org/diseases-conditions/stomach-cancer/symptoms-causes/syc-20352438
	6	https://www.cancerresearchuk.org/about-cancer/stomach-cancer/stages/stage-2
	7	https://www.cancer.net/cancer-types/stomach-cancer/symptoms-and-signs
	8	https://www.healthline.com/health/gastric-cancer
	9	https://www.cancercenter.com/cancer-types/stomach-cancer/symptoms
	10	https://www.medicinenet.com/stomach_cancer/article.htm
	11	https://www.medicalnewstoday.com/articles/257341.php
	12	https://www.independent.ie/life/health-wellbeing/health-features/botox-highly-effective-treatment-of-gastric-cancer-30524844.html
	13	https://www.cancer.ca/en/cancer-information/cancer-type/stomach/stomach-cancer/?region=on
	14	https://www.oncolink.org/cancers/gastrointestinal/gastric-cancer/all-about-gastric-cancer
	15	https://www.nhs.uk/conditions/stomach-cancer/
Yahoo n=3	16	https://www.macmillan.org.uk/information-and-support/stomach-cancer/understanding-cancer/signs-symptoms-stomach.html
	17	https://www.uclh.nhs.uk/OurServices/ServiceA-Z/Cancer/OGC/SGCAN/Pages/Home.aspx
	18	https://www.express.co.uk/life-style/health/905666/stomach-cancer-symptoms-pain-nhs
Bing n=2	19	https://www.cancer.gov/types/stomach
	20	https://www.nhsinform.scot/illnesses-and-conditions/cancer/cancer-types-in-adults/stomach-cancer

JAMA benchmark score

**Figure 2:**
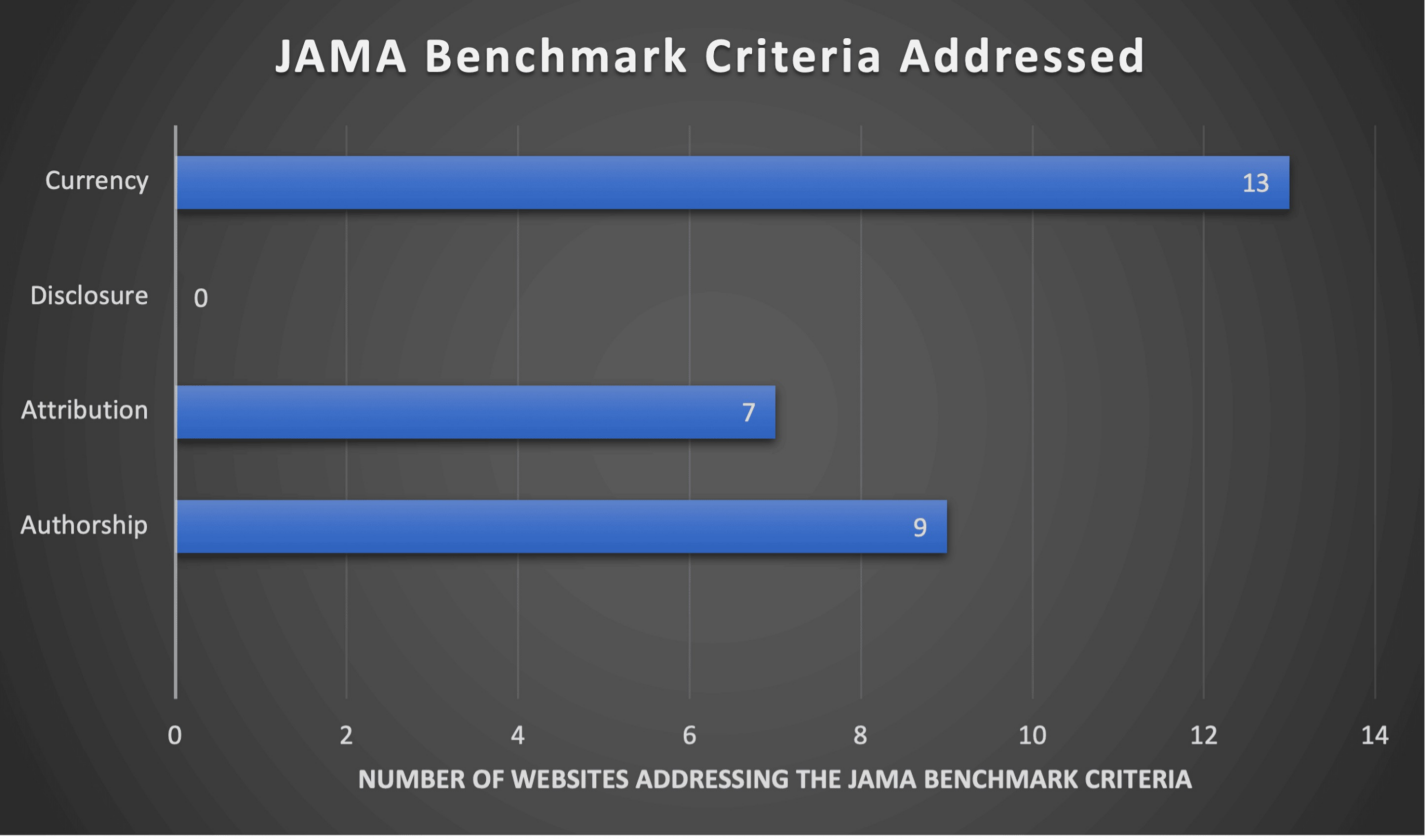
JAMA benchmark score.

The average JAMA score was 1.55. None of the twenty unique websites scored the maximum of 4 points, with two websites scoring the minimum of 0 points (21%). The most consistently addressed benchmark was Currency (date of posting and date of last review), with 14 websites addressing it (70%). The most poorly addressed benchmark was the disclosure of relevant conflicts of interest, which was addressed by none of the unique websites (0%). The breakdown of the number of websites addressing each benchmark is depicted in Figure 2.

DISCERN score

The average DISCERN score was 54.8 (68.5%), with no website achieving the maximum of 80. The highest score was 68 (85%), while the lowest was 20 (25%). The first two questions of the score concerned with the website's aims were most consistently addressed, with only one website failing to achieve the maximal 5 points (95%). Question seven, concerned with providing details of alternative sources of information and support, scored the poorest across all websites. For question 7, only one website (5%) received the maximum score of 5, whilst the remaining 19 unique websites all scored the minimum score of 1 (95%). Only eight websites had references (40%). Concerning question 16, which rates the publication's overall quality as a source of information for patients, only two websites (10%) received the maximum 5 points, representing high-quality information with minimal shortcomings. Furthermore, seven websites (35%) received the minimum score of 1 concerning question 16, reflecting low-quality information with severe or extensive shortcomings.

HON seal

**Figure 3:**
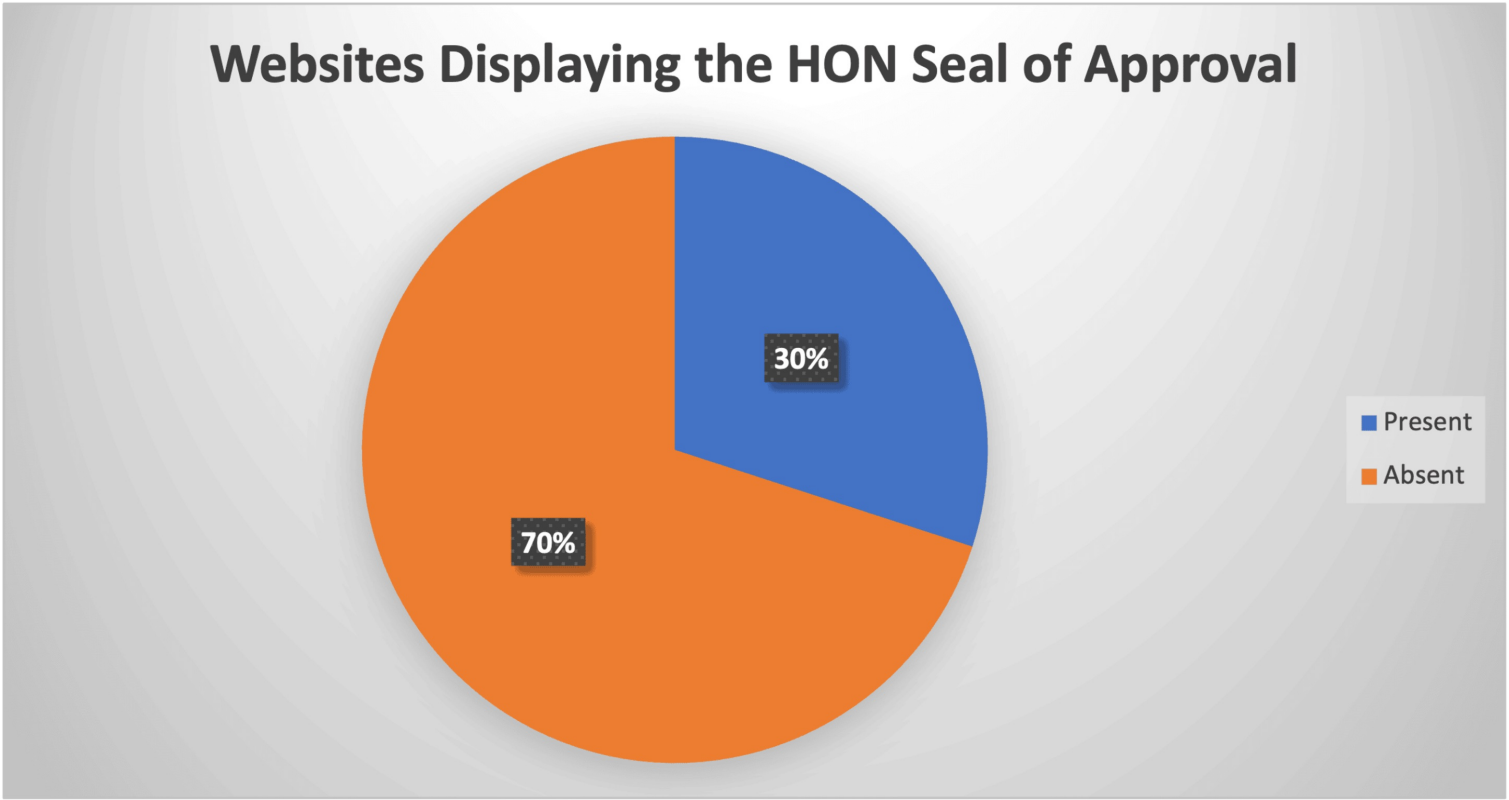
Number of websites displaying the health on the internet seal. Six websites (30%) displayed the seal, and 14 websites (70%) did not.

The HON seal was present in only six websites (30%) and absent in 14 websites (70%) (Figure 3).

GCSCS

The average GCSCS was 11 (85%). Only five websites (25%) achieved the maximum of 13 points. One website received a minimum of 0 points (0%), and another received 2 points (15%). The most poorly addressed question in the score was question 12, relating to the role of endoscopic therapy in treating early GC. This was addressed by only seven of the websites (35%). This was followed closely by question nine, relating to the details of an OGD examination, with only 12 of the unique websites discussing this detail (60%).

FRES

The average FRES was 52.7. None of the included studies obtained a score above the recommended 70. The lowest score detected was 37.9.

FKGL

The average FKGL was 9.7. Two of the included websites obtained the recommended FKGL score of 8 or less. The lowest obtained FKGL score was 5.7, and the highest was 14.1.

## Discussion

Patients are increasingly consulting the internet as their first port of call with questions related to health and well-being. This highlights the importance of ensuring that the information and websites they encounter contain high-quality and accurate information. The information contained within websites discussing health-related topics must be accurate and safe and, crucially, must advise patients when it is prudent to seek professional medical attention. As previously highlighted, this is particularly important in the case of GC. The initial signs and symptoms of GC can be subtle and often ignored by patients. Thus, any opportunity to encourage a patient to seek formal medical review must be taken.

Upon review of the results of our study, none of the 20 unique websites commented on the disclosure of conflicts of interest. Such disclosures comprise one of the four JAMA benchmarks for health-based websites. Even more concerning is that two websites scored a minimum of 0 points in the JAMA benchmark criteria, which raises serious concerns over the integrity of the information being conveyed. As highlighted previously, the JAMA benchmark criteria assess the website's integrity. So, these results would suggest severe issues with the integrity of the current websites discussing matters related to GC. This lack of integrity could lead to misinformation and potentially harm to patients.

Furthermore, seven websites (35%) achieved the minimum score of 1 in question 16 of the DISCERN score, reflecting low-quality information with severe or extensive shortcomings. Incredibly, one of the twenty unique websites scored 20 points out of a possible 80 on the DISCERN tool (25%), indicating severe deficits in the information provided. Only six of the twenty unique websites (30%) displayed the HON seal of approval and thus have signed up to the HON code of conduct and have made themselves available for regular audit of the quality of information they provide.

Interestingly, questions 10 and 11 of the DISCERN tool relate to the benefits and risks of treatment, respectively. Eighteen websites (90%) describe the benefits, but only 6 (30%) describe the risks of available treatments. Thus, most websites appear to discuss the benefits readily but downplay or do not discuss potential therapy risks. This is further evidenced by the fact that only one website (5%) addressed treatment effects on the patients' quality of life, as in question 13 of the DISCERN tool. For patients to make informed decisions about their healthcare, we feel they must be provided with all relevant information about a treatment's benefits and risks. Again, this is of particular importance to GC. The impact of a total and subtotal gastrectomy on a patient's quality of life is significant, and the patient’s expectations must be managed appropriately [14].

Concerning the GCSCS, we found that only five websites achieved a maximum of 13 points (25%), one website received a minimum of 0 points, and another website received 2 points (15%). The most poorly addressed question in the score was question 12, relating to the role of endoscopic therapy in treating early GC. This was addressed by only 7 of the websites (35%). This may reflect a lack of clinical knowledge on behalf of the authors, as the role of advanced endoscopic techniques, particularly in early GC, is ever-expanding.

The readability of the information on these websites is also of concern. First, consider the scores obtained on the FRES. The average FRES obtained was 52.7. This means the reader would require a college degree to understand the information. Indeed, none of the included websites scored the recommended 70 or greater.

The scores obtained in the FKGL were marginally better, although a significant margin of difference existed between the lowest and highest-scoring websites. Only two of the included websites attained the recommended grade level readability of 8th grade or less. The average was 9.7. The worst website obtained a grade level of 14.1.

Our findings echo previous research by Killeen et al. in their paper from 2011, in which they assessed the quality of information on the internet regarding gastric cancer. Their study highlighted similar concerns, concluding that “Internet gastric cancer information is overtly commercial, generally incomplete, and poorly accessible” [15]. Uniquely, our study has assessed both the quality of the content using the JAMA and DISCERN tools alongside our novel GCSCS and also the readability using the FRES and FKGL to give a robust description of the quality and readability of information on GC currently available on the internet.

More recently, Wang et al. [16] and Hu et al. [17] have assessed the quality of information on GC posted to various modern platforms such as TikTok and YouTube. They have found similar issues about the quality and reliability of information available.

## Conclusions

This novel study highlights the current quality of online information for patients about GC. We have identified major issues related to both the quality and readability of this information. We must educate both physicians and the public on how to recognize high-quality and reliable sources of information online. More studies must be performed to ensure that information is accurate, reliable, up-to-date, and readable for the general public.
